# Standard 6-week chemoradiation for elderly patients with newly diagnosed glioblastoma

**DOI:** 10.1038/s41598-021-01537-3

**Published:** 2021-11-11

**Authors:** Loïg Vaugier, Loïc Ah-Thiane, Maud Aumont, Emmanuel Jouglar, Mario Campone, Camille Colliard, Ludovic Doucet, Jean-Sébastien Frenel, Carole Gourmelon, Marie Robert, Stéphane-André Martin, Tanguy Riem, Vincent Roualdes, Loïc Campion, Augustin Mervoyer

**Affiliations:** 1 Department of Radiation Oncology, Institut de Cancérologie de l’Ouest (ICO), Boulevard J. Monod, 44805 Nantes-Saint-Herblain, France; 2 Department of Medical Oncology, Institut de Cancérologie de l’Ouest (ICO), Nantes-Saint-Herblain, France; 3 Department of Neurosurgery, Centre Hospitalo-Universitaire (CHU), Nantes-Saint Herblain, France; 4 Department of Biostatistics, Institut de Cancérologie de l’Ouest, St-Herblain, France; 5 Centre de Recherche en Cancérologie Nantes-Angers (CRCNA), UMR 1232 Inserm-6299 CNRS, Institut de Recherche en Santé de l’Université de Nantes, Nantes, France

**Keywords:** Cancer, Surgical oncology, Outcomes research, Cancer therapy, CNS cancer, Neurological disorders, Brain injuries, CNS cancer, Geriatrics, Prognosis, Quality of life

## Abstract

Glioblastoma (GBM) is frequent in elderly patients, but their frailty provokes debate regarding optimal treatment in general, and the standard 6-week chemoradiation (CRT) in particular, although this is the mainstay for younger patients. All patients with newly diagnosed GBM and age ≥ 70 who were referred to our institution for 6-week CRT were reviewed from 2004 to 2018. MGMT status was not available for treatment decision at that time. The primary endpoint was overall survival (OS). Secondary outcomes were progression-free survival (PFS), early adverse neurological events without neurological progression ≤ 1 month after CRT and temozolomide hematologic toxicity assessed by CTCAE v5. 128 patients were included. The median age was 74.1 (IQR: 72–77). 15% of patients were ≥ 80 years. 62.5% and 37.5% of patients fulfilled the criteria for RPA class I–II and III–IV, respectively. 81% of patients received the entire CRT and 28% completed the maintenance temozolomide. With median follow-up of 11.7 months (IQR: 6.5–17.5), median OS was 11.7 months (CI 95%: 10–13 months). Median PFS was 9.5 months (CI 95%: 9–10.5 months). 8% of patients experienced grade ≥ 3 hematologic events. 52.5% of patients without neurological progression had early adverse neurological events. Post-operative neurological disabilities and age ≥ 80 were not associated with worsened outcomes. 6-week chemoradiation was feasible for “real-life” elderly patients diagnosed with glioblastoma, even in the case of post-operative neurological disabilities. Old does not necessarily mean worse.

## Introduction

Glioblastoma (GBM) is the most malignant and common primary brain tumor in adults^1^. The overall prognosis remains poor: around 12–15 months^2,3^. The current therapeutics rely on surgical resection, radiotherapy (RT), chemotherapy (CT) and best supportive cares (BSC)^4^. The combination of 6 weeks of conventionally fractionated RT (CFRT) with radiosensitizing temozolomide (TMZ), followed by up to 6 months of maintenance TMZ (known as the standard chemoradiation regimen^3^) is the mainstay for < 65 year-old patients^4^. However, there are concerns about whether and/or which elderly patients may benefit from such post-operative treatment.

Focusing on the elderly population (e.g. aged ≥ 70 years) with newly-diagnosed GBM is relevant for the following reasons: (i) the highest incidence rate is currently observed in patients aged 75–84 years^5^; (ii) neurological symptoms (following progression or treatment toxicities) may have dramatic consequences on independence and/or quality of life for such a frail population^6,7^; (iii) the life expectancy is extremely poor but has increased in the last decade with the development of post-operative treatments^2^; (iv) GBM-specific geriatric scales of frailty are still lacking. Age and performance status (PS) > 2 are common negative prognostic factors^8,9^. MGMT (O6-methylguanine-DNA methyltransferase) DNA-repair gene silencing and its consequence on therapeutics have been investigated considerably^10,11^. In particular, MGMT methylation is associated with improved response to TMZ. The difficulties in interpreting the results of the tests nevertheless mean that MGMT status not routinely assessed^12^.

Over the last few years, both the indication for, and modalities of, post-operative treatments in elderly patients have been controversial^13–16^. Patients > 70 years were not included in the original study by Stupp et al.^3^ but they may benefit from the standard chemoradiation regimen compared to RT alone, particularly in case of PS ≤ 1 and macroscopically complete surgical resection^17–26^. However, it is important to note that for such population, post-operative RT could also result in only modest improvements compared to BSC and despite a Karnofsky index > 70, whereas the duration of the CFRT may represent almost one third of their life expectancy^27^. Hypofractionated and accelerated RT protocols over 1 or 3 weeks (HFRT) have emerged in this context, with outcomes comparable with those of CFRT and acceptable tolerance^28–32^. Recently, a large phase 3 trial has shown the survival benefits of HFRT with TMZ versus HFRT alone for > 65 year-old and PS ≤ 2 patients^33^. This type of regimen tends to be the current standard of care for elderly patients although there are no prospective trials comparing it to the standard 6-week chemoradiation. Interestingly, two ongoing phase 3 trials: EORTC-1709-BTG (NCT03345095) (RT + TMZ and marizomib); and RT “dose painting” escalation + TMZ (SPECTRO-GLIO, NCT01507506)^34^, have contributed to bringing to the fore the standard 6-week chemoradiation regimen for patients with no upper limit of age.

In this context, we present the tolerance data and outcomes for all the elderly patients (≥ 70 years) who were referred to our institution for the standard 6-week chemoradiation. The objective was to investigate whether common geriatric sources of frailty such as age or baseline neurological disabilities, had a negative impact on survival.

## Materials and methods

### Patient selection

All ≥ 70-year-old, histologically-proven GBM patients referred to our radiation therapy department (Institut de Cancérologie de l’Ouest, Saint Herblain, France) for a standard 6-week chemoradiation from January 2004 to December 2018 were included. Patients with World Health Organisation (WHO) grade < 4 gliomas were excluded^1^. All patients had surgical intervention—either complete (CR) or partial (PR) resection or biopsy (B)—and had been considered able to tolerate 6-week chemoradiation (CRT) by a multidisciplinary team (including neurosurgeons, medical and radiation oncologists), mainly based on PS ≤ 2. Specific geriatric evaluation was not systematically performed in our institution at that time. The histomolecular isocitrate deshydrogenase (IDH) mutation was determined in each case, but after 2011. MGMT-methylation status was not considered informative for therapeutic decisions in our institution at that time and was not carried out routinely.

### Post-operative treatment modalities and follow-up

Immobilization in the treatment position was systematically achieved using custom thermoplastik mask contention during RT. An RT-dedicated computed tomography (CT) scan was registered with contrast-enhanced T1-weighted brain magnetic resonance imaging (MRI) in order to guide tumor delineation^35,36^. The gross tumor volume (GTV) was defined as the contrast enhancement area in the T1-weighted MRI sequence and CT scan, including the tumor bed for patients with prior partial or complete resection. Following GBM guidelines^36^, the clinical target volume (CTV) was defined as the addition of a geometric tridimensional 10–20 mm margin (depending on the tumor’s topography) around the GTV that was corrected to the anatomical borders and had to include the hypersignal FLAIR-MRI around the GTV. The planning target volume (PTV) was defined as CTV + 5 mm. The dose prescribed to the PTV was 60 Gy in 30 fractions of 2 Gy per fraction, 5 days a week within conformal three-dimensional radiotherapy^3^. Concomitant daily TMZ (75 mg/m^2^, 7 days a week from the first to the last day of RT) was prescribed during RT, with weekly blood samples.

All patients were examined by their medical oncologists 1 month after the last RT session to start up to 6 cycles of maintenance TMZ (150–200 mg/m^2^, 5 consecutive days a month). During the CRT, treatment tolerance was evaluated once a week. Patients were followed up clinically and with blood tests once a month throughout the maintenance phase and then every 3 months. The first brain MRI for evaluation was performed 3 months after the end of RT, then every 3 months for at least 5 years.

### Outcomes

The primary endpoint was overall survival (OS). Secondary endpoints were progression-free survival (PFS), incidence rate for early adverse neurological events, and TMZ-related toxicity assessed by the CTCAE v5 classification.

Survivals (OS and PFS) were respectively defined as the time from histological diagnosis to death from any cause, and neurological progression as assessed by MRI (at least T1 with gadolinium injection and FLAIR) or death from any cause. As it can be difficult to distinguish recurrence from pseudoprogression after RT and TMZ^37^, repeated MRI over shorter time interval than planned were necessary in some cases. The date of progression assigned was the earlier date when progression was first suspected. Early adverse neurological events were defined as the occurrence of: symptoms of intracranial hypertension (ICHT) and/or the use of corticosteroids and/or the need for hospitalization for any cause in the absence of neurological progression or death (≤ 1 month after CRT).

For each patient, the presence of neurological disabilities (motor, visual, instability, cognitive and communication) was also retrospectively reviewed before and after CRT. Motor disabilities were scored between mild (e.g. paresis) and severe (e.g. objective neurological deficit), since grading with the Medical Research Council (MRC) scale was not retrospectively feasible. Visual disabilities corresponded to homonymous hemianopias or anopsias. Instability included dizziness and proprioceptive disorders; cognitive disabilities included disorientation in time and place, frontal syndrome, executive functioning or mnesic disorders.

### Statistical analysis

Qualitative factors were described in terms of the frequency of their respective modalities and compared using of Pearson’s Chi-square test (or Fisher test). For continuous factors, independent groups were described by means of their median [range] and compared using a Student’s *t* test (or Mann–Whitney). Survival (PFS and OS) was described by means of Kaplan–Meier curves and compared between interest groups using log-rank tests. Median follow-up was calculated by means of inverse Kaplan–Meier method. Univariate logistic regressions were performed to assess prognostic factors on the occurrence of adverse neurological events. The RPA class by Scott et al.^8^—I (II): ≤ (>) 75.5 with CR/PR; III (IV): PS ≤ (>) 1 with B—was considered. All tests were two-sided; significance was set at p = 0.05, all calculations were made using Stata 16.1 SE (StataCorp LLC, College Station, Texas, USA).

### Ethics approval

This study was approved by the ethic committee of the Centre Hospitalo-Universitaire (CHU) in Angers, France (Number 2020/117). Our institution has an approved and standardized informed consent process that includes research and access to data. All patients were informed to ensure their non-opposition to the use of their data for research purposes and informed consent has been obtained from all participants. We have received an authorization by Ethics Committee of Angers Hospital to retrospectively analyse and use patients data. All the study and analysis were performed in accordance with the relevant guidelines and regulations.

## Results

A cohort of 128 patients was established from January 2004 to December 2018. Patient characteristics are summarized in Table 1. All patients after 2011 had IDH wild-type GBM. The median age was 74.1 (IQR: 72–77) with 73/128 (57%) male. Most patients had PS 0 (49/128; 38.5%) and 1 (71/128; 55.5%). 39%, 23.5%, 33.5% and 4% patients respectively fulfilled the criteria for RPA class I, II, III and IV. 19/128 (15%) were ≥ 80 years old, with 11 RPA II, 5 RPA III and 3 RPA IV.

**Table 1:** Patient and treatment characteristics (N = 128). Several patients with pre-chemoradiation (CRT) neurological deficits had more than one disability.

**Gender**	
Male	73 (57%)
Female	55 (43%)
**Median age [range] (years)**	74 [70–88]
70–75.5	73 (57%)
≥ 75.5	55 (43%)
**Performance status**	
0	49 (38.5%)
1	71 (55.5%)
2	7 (5.5%)
3	1 (1%)
**Type of surgery**	
Complete resection	64 (50%)
Partial resection	17 (13.5%)
Biopsy	47 (36.5%)
**RPA class**	
I	50 (39%)
II	30 (23.5%)
III	43 (33.5%)
IV	5 (4%)
**Pre-CRT neurological disabilities**	76 (59.5%)
Mild motor disabilities	26 (20.5%)
Severe motor disabilities	6 (4.5%)
Visual disabilities^†^	17 (13.5%)
Instabilities^‡^	6 (4.5%)
Cognitive disabilities^‡‡^	10 (8%)
Communication disorders	28 (22%)

^†^E.g. homonymous hemianopias or anopsias.

^‡^E.g. dizziness or proprioceptive disorders.

^‡‡^E.g. disorientation with time and place, frontal syndrome, executive functioning or mnesic disorders.

104 patients (81%) received the entire 6-week CRT and 36 (28%) completed the further 6 maintenance TMZ months (Table 2). 13 (10%) patients did not fulfill the 30 RT fractions because of major overall worsening or death; 11 (9%) patients received 60 Gy but < 6 concomitant TMZ weeks because of blood toxicity or swallowing troubles for one patient. 91 patients (71%) received < 6 maintenance TMZ months because of death or progression. The median number for RT fractions, concomitant TMZ weeks and maintenance TMZ months were respectively 30 (IQR, 30–30), 6 (IQR, 6–6) and 2 (IQR, 0–6). All ≥ 80-year-old patients received the 30 RT fractions.

**Table 2:** Treatment characteristics (N = 128). *Pts* patients, *RT* radiotherapy, *TMZ* temozolomide.

Pts having received 30 RT fractions	115 (90%)
Pts having received 6 concomitant TMZ weeks	107 (83.5%)
Pts having received 6 maintenance TMZ months	36 (28%)

The rate for grade ≥ 3 TMZ-induced blood toxicity (mainly thrombopenia) yielded 8% (10/128). 58.5% (75/128) and 57% (73/128) had pre- and post-CRT neurological disabilities, of whom 4.5% (6/128) were severe prior to the CRT. One patient had fully regressive facial paralysis after treatment, while the neurological symptoms were stable for the other five. Neither age ≥ 80 (p = 0.21), B (p = 0.22), III–IV RPA class (p = 0.19) nor pre-CRT neurological disabilities (p = 0.86), were associated with incomplete CRT.

Of the 84 patients (65.5%; 84/128) alive and harboring neurological progression, 51/84 (60.7%) and 33/84 (39.3%) patients had respectively a second line of chemotherapy and BSC following progression.

### Follow-up and survivals

**Figure 1:**
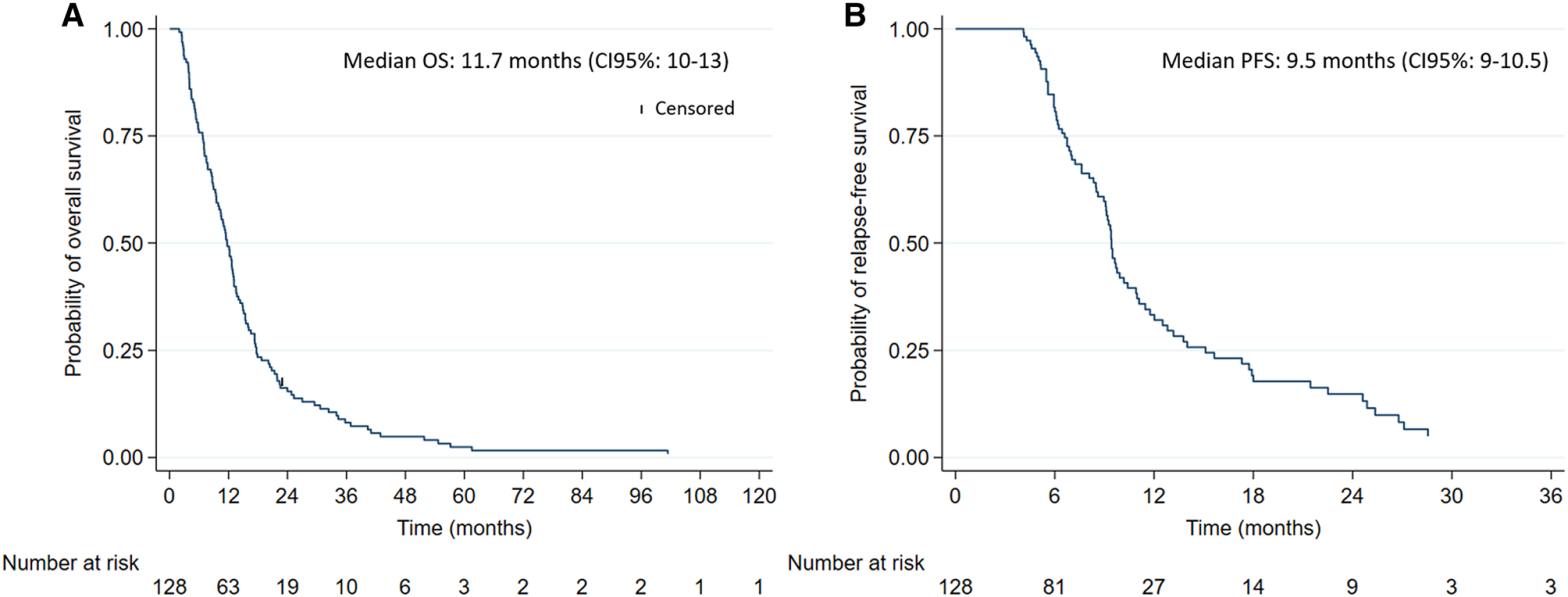
(**A**) Overall survival (OS) and (**B**) progression-free survival (PFS). Median follow-up: 11.7 months (IQR: 6.5–17.5). All patients but 2 had died at the time of analysis: one patient was censored after progression at 20 months and one patient was still alive at 120 months.

**Figure 2:**
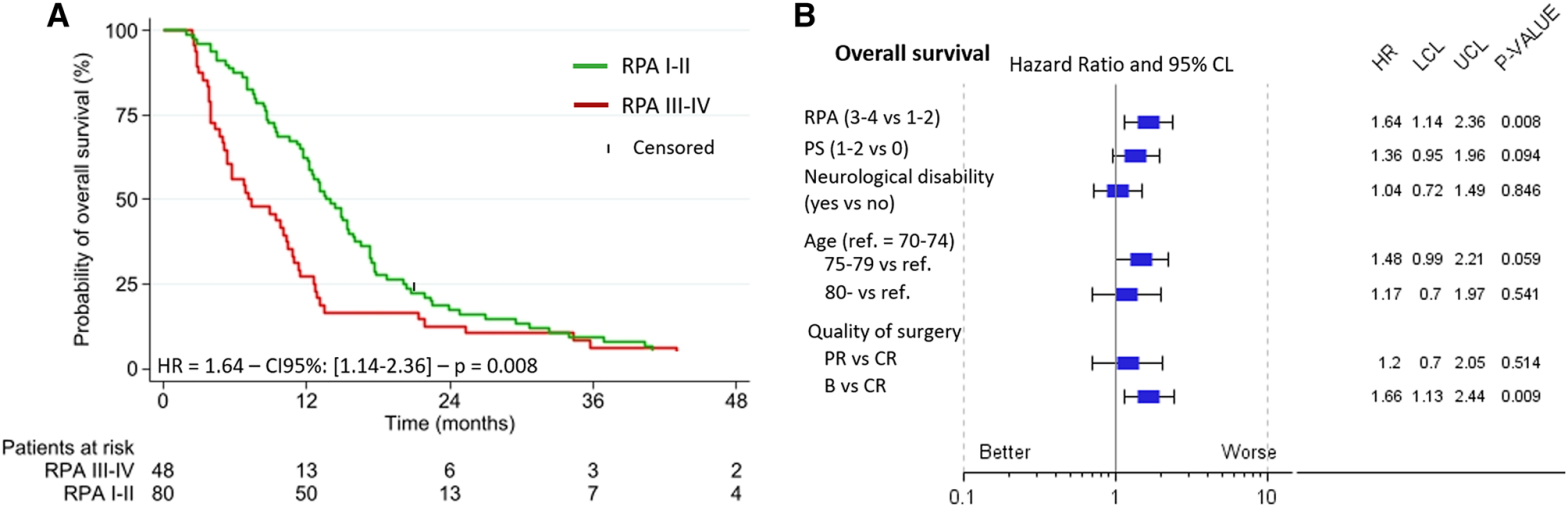
(**A**) Overall survival (OS) depending on the RPA class and (**B**) OS forest plot in univariate analysis. *PS* performance status, *C*(*P*)*R* complete (partial) resection, *B* biopsy. Neurological disability = pre-chemoradiation motor, visual, instability, cognitive or communication disability.

All patients but 2 had died at the time of analysis. Oncologic outcomes are summarized in Table 3 and Figs. 1 and 2. With a median follow-up of 11.7 months (IQR: 6.5–17.5), the median OS was 11.7 months (CI 95%: 10–13 months). The 2- and 5-year OS was 15% (CI 95%: 10–22%) and 2.4% (CI 95%: 0.6–6%), respectively. The median PFS was 9.5 months (CI 95%: 9–10.5 months). 19.5% (25/128) of patients had early neurological progression or death (either during or ≤ 1 month after CRT). The median OS for patients who completed 6-week CRT (N = 104) was: 12.5 months (CI 95%: 10.8–13.5), and who did not complete 6-week CRT (N = 24): 3.4 months (CI 95%: 2.5–4.1). For the patients who had the entire 6-week CRT and 6 maintenance TMZ months (N = 36), the median OS was 17.5 months (CI 95%: 15.4–30.6).

**Table 3:** Progression-free and overall survival outcomes (N = 128).

**Progression-free survival**	
Median (months)	9.4 (CI 95%: 8.9–10.4)
1-year	33.3% (CI 95%: 23.8–43.1)
**Overall survival**	
Median (months)	11.7 (CI 95%: 9.9–13.1)
1-year	49.2% (CI 95%: 40.3–57.5)
2-year	15.4% (CI 95%: 9.8–22.2)
5-year	2.4% (CI 95%: 0.6–6.0)

The quality of the surgical resection (B versus CR; HR = 1.66, p = 0.009) and RPA class (III–IV versus I–II; HR = 1.64, p = 0.008) were significantly associated with death from any cause in univariate analysis but this was not the case for either age ≥ 80 (HR = 1.17, p = 0.54) or presence of pre-CRT neurological disabilities (HR = 1.04, p = 0.84) (Fig. 2).

### Subgroup of ≥ 80 year-old patients

Regarding the ≥ 80-year-old cohort, the median OS and PFS were 12.1 and 9.2 months, respectively. Only one ≥ 80-year-old patient (5.5%; 1/19) had early neurological progression. The quality of the surgical resection (B-PR vs CR: HR = 2.79, p = 0.045) was significantly associated with death but this was not the case for neither RPA class (III–IV versus II; HR = 2.16, p = 0.127) nor presence of pre-CRT neurological disabilities (HR = 1.21, p = 0.688).

### Early adverse neurological events

In the subgroup of patients without early neurological progression or death (80.5%; 103/128), 52.5% (54/103) of patients had early adverse neurological events. The occurrence of such events (HR = 1.69, p = 0.010) was significantly associated with death from any cause in this subgroup of patients. Regarding specifically the ≥ 80-year-old cohort, the rate for early adverse neurological events was 61% (11/18).

**Figure 3:**
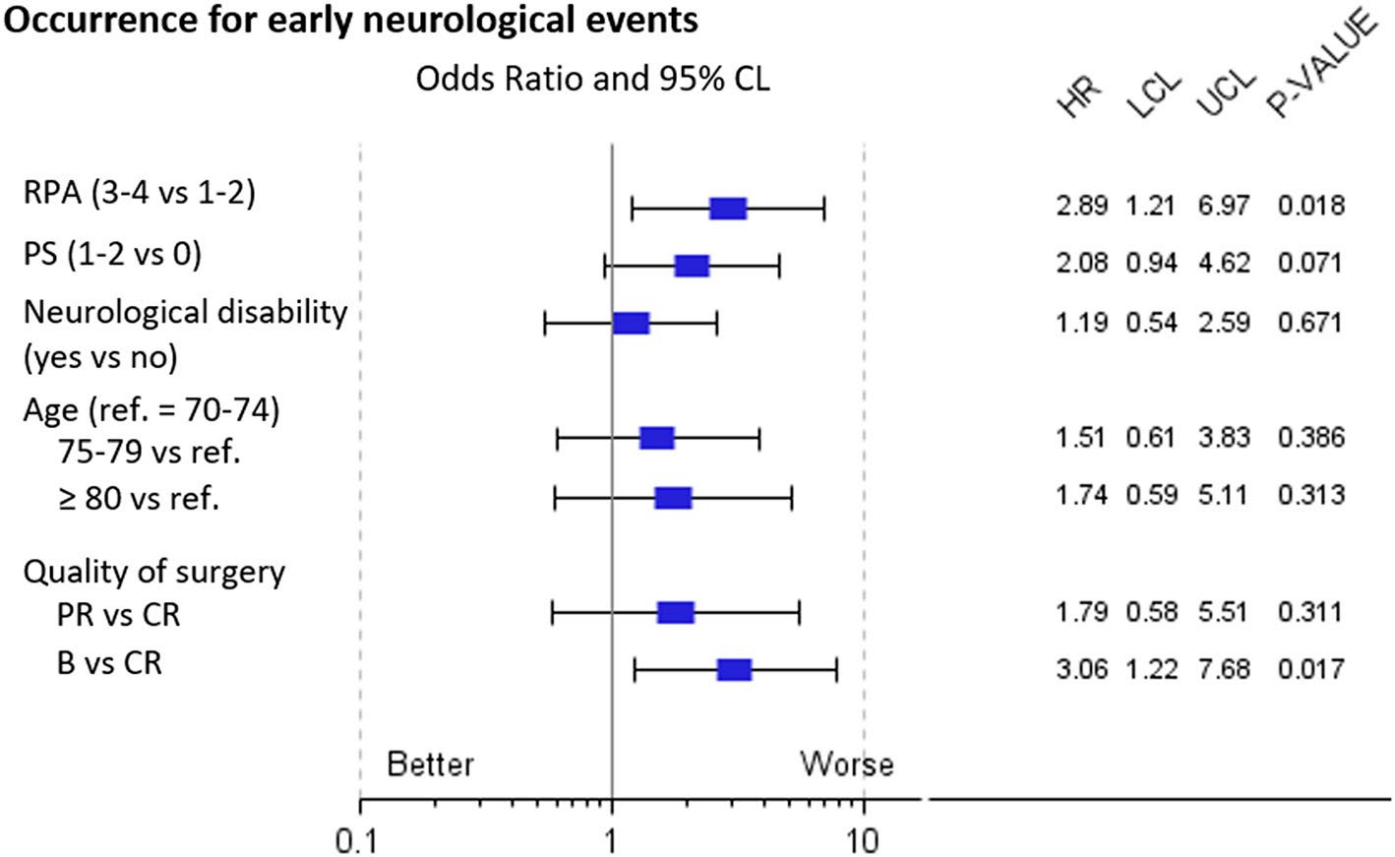
Forest plot in univariate analysis for the occurrence of early adverse neurological events (defined as the occurrence of intracranial hypertension symptoms and/or use of corticosteroids and/or hospitalization) for the subgroup of patients without neurological progression or death before the start of temozolomide maintenance (N = 103). *CRT* chemoradiation, *PS* performance status, *C*(*P*)*R* complete (partial) resection, *B* biopsy. Neurological disability = pre-CRT motor, visual, instability, cognitive or communication disability.

Prognostic factors in the same subgroup (N = 103) for the occurrence of early adverse neurological events are summarized in Fig. 3. Patients with pre-CRT neurological disabilities did not exhibit significantly higher occurrence for early adverse neurological events (OR = 1.19, p = 0.671) nor did ≥ 80-year-old patients (OR = 1.74, p = 0.313). The quality of the surgical resection (B versus CR; OR = 3.06, p = 0.017) and RPA class (III–IV versus I–II, OR = 2.89, p = 0.018) were significantly associated with higher incidence for such events.

## Discussion

Around 80% of all the ≥ 70-year-old patients who were referred to our institution for standard 6-week chemoradiation, received the entire 6-week long treatment. In particular, all the ≥ 80-year-old patients completed this treatment. The RPA classification by Scott et al.^8^ was prognostic for both the OS and occurrence of early adverse neurological events, but interestingly, the presence of neurologic disabilities at baseline was not associated with worsened outcomes.

In recent decades, several studies have investigated different treatment modalities to go further than the standard protocol: e.g. CT intensification with lomustine for MGMT-methylated patients^38^, TMZ dose escalation^39^ or the addition of irinotecan during the maintenance TMZ phase^40^; maintenance TMZ beyond 6 cycles^41^; the addition of antiangiogenic drugs such as bevacizumab^42,43^ or cilengitide^44^; immunotherapeutic approaches with vaccines such as Rindopepimut^®^ for patients with a mutation in the epidermal growth factor receptor (EGFR) gene^45^ or antiPD1 checkpoint inhibitors^46^; alternating electric fields to the brain called Tumor Treating Fields (TTF) during the maintenance phase^47^. At this time, none of these treatments except TTF has been able to demonstrate clear oncologic improvements compared to the original 6-week chemoradiation regimen, which remains the mainstay for ≤ 65–70-year-old patients with median PFS and OS of 6.9 and 14.6 months, respectively^3^. In comparison (Table 4), median OS were 3.9–9.6 months with exclusive RT or TMZ^28,30,33,48^. It is important to note that standard 6-week chemoradiation has never been formally compared with hypofractionated chemoradiation. Considering overall survival (MGMT methylated and unmethylated combined), the data of our study (median OS 11.7 months) compare favorably to the data by Perry et al.^33^ (median OS 9.3 months), with also comparable median age (74.1 years in our study versus 73 years). Whereas age commonly acts as an obstacle for the standard 6-week chemoradiation, the results of our study show that (i) standard CRT (> 80% completion rate) was feasible for ≥ 70 and even ≥ 80-year-old GBM patients; (ii) the survival rates (regardless of MGMT status) were rather comparable to the values for trial-selected and/or younger patients. Similar prospective/retrospective analyses have already corroborated this observation^18–26^.

**Table 4:** Median overall survival (OS) from prospective randomized trials with different post-operative modalities compared to our study. *CFRT* conventionally fractionated RT, *HFRT* hypofractionated RT, *TMZ* temozolomide, *SD* standard deviation, *NS* not specified.

	Our study	Perry^33^	Malmström^28^	Wick^†^^48^	Roa^30^
Median (range) age (years)	74.1 (70–88)	73 (65–90)	70 (60–88)	71 (66–84)	72 ± 5.5 (SD)
**Median (CI 95%) OS (months)**					
CFRT + TMZ	11.7 (9.9–13.1)	–	–	–	–
CFRT	–	–	6 (5.1–6.8)	9.6 (8.2–10.8)	5.1 (NS)
HFRT + TMZ	–	9.3 (8.3–10.3)	–	–	–
HFRT	–	7.6 (7.0–8.4)	7.5 (6.5–8.6)	–	5.6 (NS)
TMZ	–	–	8.3 (7.1–8.5)	8.6 (7.3–10.2)	–

^†^Patients with malignant anaplastic astrocytoma were also included.

Neurological deficits in elderly patients with GBM are sometimes used as a reason to avoid “aggressive” post-operative treatment. But this cohort of patients tolerated the treatment reasonably well. The presence of neurological disability at baseline was associated with neither worsened OS nor higher occurrence of early adverse neurological events. The presence of baseline neurological disability may reflect the extent of the surgical resection, and the survival was clearly improved in patients who had either a CR or a PR, even for those ≥ 80 year-old. Although often considered as a source of geriatric frailty, the presence of neurological disability alone should not be a reason for post-operative de-escalation.

Lastly, the treatment modalities and duration of treatment for the standard chemoradiation may respectively seem too heavy and too long compared to the life expectancy of elderly patients with GBM, but the overall survival in this study was actually similar to the life expectancy of younger patients with GBM. A surprising 81% of these elderly patients completed 6-week CRT. Only 28% of patients completed the full 6 cycles of maintenance temozolomide. Around 50% of patients developed early adverse neurological events, which were correlated with lower OS as already described in the literature^9^. In comparison in the EORTC/CCTG trial^33^, the completion rate for 3-week RT and the median number of TMZ cycles were 100% and 5, respectively. Various therapeutic options specifically aimed at the elderly have emerged in this regard. Accelerated HFRT ± TMZ is increasingly being used with significantly lower radiation doses but paradoxically comparable outcomes^28–33^. TMZ monotherapy is also an option in the case of MGMT methylation^10–12,28,48,49^. HFRT without TMZ and TMZ monotherapy were even shown to improve OS compared to 6-week RT in the Nordic randomized phase 3 trial for ≥ 70 year-old patients^28^. The fear of therapeutic de-escalation arising from such protocols means they are not recommended for younger patients^26^. Some elderly patients could however appear suitable for the best and maybe most “aggressive” strategy, but reliable predictive biomarkers are lacking in order to identify which elderly patients would fit into this category. The development of GBM-dedicated geriatric scales e.g. relying on the RPA classification appears crucial for optimizing treatment algorithms^13,14^.

Our study has obvious limitations, mainly linked to its retrospective nature and the selection bias. The criteria for the patient selection in our study (i.e. based on the referral to our department for standard 6-week chemoradiation) may seem unsatisfactory since not all the patients ≥ 70 years with GBM were thus analyzed. All the patients included have been considered able to tolerate 6-week chemoradiation mainly based on PS < 2 and before the implementation of a systematic geriatric assessment in our department. Nutrition and mood assessments are other important geriatric parameters, but the data were incomplete or missing from our recording. Specific evaluation of quality of life as based on patient-reported outcomes, is also missing. Specific response assessment criteria in neuro-oncology (RANO) have been developed for GBM^50^ but could not be used in our study because of the retrospective analysis. Overall prospective geriatric evaluation is needed to build GBM-dedicated treatment algorithms.

## Conclusions

Standard 6-week chemoradiation was feasible for “real-life” elderly patients diagnosed with glioblastoma with unknown MGMT status, even in cases of post-operative neurological disabilities. GBM-dedicated geriatric scales are urgently needed to guide optimal therapeutics.

## Data Availability

Research data are stored in an institutional repository and will be shared upon request to the corresponding author.

## References

[1] Ricard D, Idbaih A, Ducray F, Lahutte M, Hoang-Xuan K, Delattre J-Y (2012). Primary brain tumours in adults. Lancet.

[2] Shah BK, Bista A, Sharma S (2016). Survival trends in elderly patients with glioblastoma in the United States: A population-based study. Anticancer Res..

[3] Stupp R (2005). Radiotherapy plus concomitant and adjuvant temozolomide for glioblastoma. N. Engl. J. Med..

[4] Weller M (2017). European Association for Neuro-Oncology (EANO) guideline on the diagnosis and treatment of adult astrocytic and oligodendroglial gliomas. Lancet Oncol..

[5] Ostrom QT, Gittleman H, Truitt G, Boscia A, Kruchko C, Barnholtz-Sloan JS (2018). CBTRUS Statistical Report: Primary brain and other central nervous system tumors diagnosed in the United States in 2011–2015. Neuro-oncology.

[6] Lorimer CF, Saran F, Chalmers AJ, Brock J (2016). Glioblastoma in the elderly—How do we choose who to treat?. J. Geriatr. Oncol..

[7] Hoffe S, Balducci L (2012). Cancer and age: General considerations. Clin. Geriatr. Med..

[8] Scott JG (2012). Recursive partitioning analysis of prognostic factors for glioblastoma patients aged 70 years or older. Cancer.

[9] Rahman R (2015). Incidence, risk factors, and reasons for hospitalization among glioblastoma patients receiving chemoradiation. J. Neurooncol..

[10] Hegi ME (2005). MGMT gene silencing and benefit from temozolomide in glioblastoma. N. Engl. J. Med..

[11] Gerstner ER, Yip S, Wang DL, Louis DN, Iafrate AJ, Batchelor TT (2009). MGMT methylation is a prognostic biomarker in elderly patients with newly diagnosed glioblastoma. Neurology.

[12] Hegi ME (2019). MGMT promoter methylation cutoff with safety margin for selecting glioblastoma patients into trials omitting temozolomide: A pooled analysis of four clinical trials. Clin. Cancer Res..

[13] Jordan JT, Gerstner ER, Batchelor TT, Cahill DP, Plotkin SR (2016). Glioblastoma care in the elderly. Cancer.

[14] Asmaa A (2018). Management of elderly patients with glioblastoma-multiforme—A systematic review. Br. J. Radiol..

[15] Minniti G, Lombardi G, Paolini S (2019). Glioblastoma in elderly patients: Current management and future perspectives. Cancers.

[16] Fiorentino A (2015). Can elderly patients with newly diagnosed glioblastoma be enrolled in radiochemotherapy trials?. Am. J. Clin. Oncol..

[17] Stupp R (2009). Effects of radiotherapy with concomitant and adjuvant temozolomide versus radiotherapy alone on survival in glioblastoma in a randomised phase III study: 5-year analysis of the EORTC-NCIC trial. Lancet Oncol..

[18] Gzell C, Wheeler H, Guo L, Kastelan M, Back M (2014). Elderly patients aged 65–75 years with glioblastoma multiforme may benefit from long course radiation therapy with temozolomide. J. Neurooncol..

[19] Franceschi E (2016). Which elderly newly diagnosed glioblastoma patients can benefit from radiotherapy and temozolomide? A PERNO prospective study. J. Neurooncol..

[20] Minniti G (2008). Radiotherapy plus concomitant and adjuvant temozolomide for glioblastoma in elderly patients. J. Neurooncol..

[21] Barker CA (2012). Radiotherapy and concomitant temozolomide may improve survival of elderly patients with glioblastoma. J. Neurooncol..

[22] Biau J (2017). Radiotherapy plus temozolomide in elderly patients with glioblastoma: A ‘real-life’ report. Radiat. Oncol..

[23] Chang-Halpenny CN, Yeh J, Lien WW (2015). Elderly patients with glioblastoma multiforme treated with concurrent temozolomide and standard- versus abbreviated-course radiotherapy. Perm. J..

[24] Combs SE (2008). Postoperative treatment of primary glioblastoma multiforme with radiation and concomitant temozolomide in elderly patients. Int. J. Radiat. Oncol. Biol. Phys..

[25] Gerstein J (2010). Postoperative radiotherapy and concomitant temozolomide for elderly patients with glioblastoma. Radiother. Oncol..

[26] Hanna C (2020). Treatment of newly diagnosed glioblastoma in the elderly: A network meta-analysis. Cochrane Database Syst. Rev..

[27] Keime-Guibert F (2007). Radiotherapy for glioblastoma in the elderly. N. Engl. J. Med..

[28] Malmström A (2012). Temozolomide versus standard 6-week radiotherapy versus hypofractionated radiotherapy in patients older than 60 years with glioblastoma: The Nordic randomised, phase 3 trial. Lancet Oncol..

[29] Roa W (2015). International atomic energy agency randomized phase III study of radiation therapy in elderly and/or frail patients with newly diagnosed glioblastoma multiforme. J. Clin. Oncol..

[30] Roa W (2004). Abbreviated course of radiation therapy in older patients with glioblastoma multiforme: A prospective randomized clinical trial. J. Clin. Oncol..

[31] Arvold ND (2015). Hypofractionated versus standard radiation therapy with or without temozolomide for older glioblastoma patients. Int. J. Radiat. Oncol. Biol. Phys..

[32] Minniti G (2015). Standard (60 Gy) or short-course (40 Gy) irradiation plus concomitant and adjuvant temozolomide for elderly patients with glioblastoma: A propensity-matched analysis. Int. J. Radiat. Oncol. Biol. Phys..

[33] Perry JR (2017). Short-course radiation plus temozolomide in elderly patients with glioblastoma. N. Engl. J. Med..

[34] Laprie A (2019). Dose-painting multicenter phase III trial in newly diagnosed glioblastoma: The SPECTRO-GLIO trial comparing arm A standard radiochemotherapy to arm B radiochemotherapy with simultaneous integrated boost guided by MR spectroscopic imaging. BMC Cancer.

[35] Sulman EP (2017). Radiation therapy for glioblastoma: American Society of Clinical Oncology clinical practice guideline endorsement of the American Society for Radiation Oncology guideline. J. Clin. Oncol..

[36] Feuvret L, Antoni D, Biau J, Truc G, Noël G, Mazeron J-J (2016). Guidelines for the radiotherapy of gliomas. Cancer Radiother..

[37] Verma N, Cowperthwaite MC, Burnett MG, Markey MK (2013). Differentiating tumor recurrence from treatment necrosis: A review of neuro-oncologic imaging strategies. Neuro Oncol..

[38] Herrlinger U (2019). Lomustine-temozolomide combination therapy versus standard temozolomide therapy in patients with newly diagnosed glioblastoma with methylated MGMT promoter (CeTeG/NOA-09): A randomised, open-label, phase 3 trial. Lancet.

[39] Gilbert MR (2013). Dose-dense temozolomide for newly diagnosed glioblastoma: A randomized phase III clinical trial. J. Clin. Oncol..

[40] Lieberman FS (2019). Phase 2 study of radiation therapy plus low-dose temozolomide followed by temozolomide and irinotecan for glioblastoma: NRG Oncology RTOG Trial 0420. Int. J. Radiat. Oncol. Biol. Phys..

[41] Blumenthal DT (2017). Is more better? The impact of extended adjuvant temozolomide in newly diagnosed glioblastoma: A secondary analysis of EORTC and NRG Oncology/RTOG. Neuro-oncology.

[42] Gilbert MR (2014). A randomized trial of bevacizumab for newly diagnosed glioblastoma. N. Engl. J. Med..

[43] Chinot OL (2014). Bevacizumab plus radiotherapy-temozolomide for newly diagnosed glioblastoma. N. Engl. J. Med..

[44] Stupp R (2014). Cilengitide combined with standard treatment for patients with newly diagnosed glioblastoma with methylated MGMT promoter (CENTRIC EORTC 26071–22072 study): A multicentre, randomised, open-label, phase 3 trial. Lancet Oncol..

[45] Weller M (2017). Rindopepimut with temozolomide for patients with newly diagnosed, EGFRvIII-expressing glioblastoma (ACT IV): A randomised, double-blind, international phase 3 trial. Lancet Oncol..

[46] Lim M, Xia Y, Bettegowda C, Weller M (2018). Current state of immunotherapy for glioblastoma. Nat. Rev. Clin. Oncol..

[47] Stupp R (2017). Effect of tumor-treating fields plus maintenance temozolomide vs maintenance temozolomide alone on survival in patients with glioblastoma: A randomized clinical trial. JAMA.

[48] Wick W (2012). Temozolomide chemotherapy alone versus radiotherapy alone for malignant astrocytoma in the elderly: The NOA-08 randomised, phase 3 trial. Lancet Oncol..

[49] Gállego Pérez-Larraya J (2011). Temozolomide in elderly patients with newly diagnosed glioblastoma and poor performance status: An ANOCEF phase II trial. J. Clin. Oncol..

[50] Ellingson BM, Wen PY, Cloughesy TF (2017). Modified criteria for radiographic response assessment in glioblastoma clinical trials. Neurotherapeutics.

